# Mesenchymal Stromal Cells Do Not Increase the Risk of Viral Reactivation Nor the Severity of Viral Events in Recipients of Allogeneic Stem Cell Transplantation

**DOI:** 10.1155/2012/690236

**Published:** 2012-05-30

**Authors:** Giovanna Lucchini, Erica Dander, Fabio Pavan, Irene Di Ceglie, Adriana Balduzzi, Paolo Perseghin, Giuseppe Gaipa, Alessandra Algarotti, Martino Introna, Alessandro Rambaldi, Attilio Rovelli, Andrea Biondi, Ettore Biagi, Giovanna D'Amico

**Affiliations:** ^1^Clinica Pediatrica, Università degli Studi di Milano Bicocca, Ospedale San Gerardo, 20900 Monza, Italy; ^2^Centro Ricerca “M. Tettamanti”, Clinica Pediatrica, Università degli Studi di Milano Bicocca, 20900 Monza, Italy; ^3^Servizio Trasfusionale, Unità di Aferesi e Criobiologia, Ospedale San Gerardo, 20900 Monza, Italy; ^4^Laboratorio di Terapia Cellulare “Stefano Verri”, Ospedale San Gerardo, 20900 Monza, Italy; ^5^Unità Strutturale Complessa di Ematologia, Ospedali Riuniti di Bergamo, 24128 Bergamo, Italy

## Abstract

Mesenchymal stromal cells (MSC) are tested in clinical trials to treat graft versus host disease (GvHD) after stem cell transplantation (SCT). *In vitro* studies demonstrated MSC's broad immunosuppressive activity. As infections represent a major risk after SCT, it is important to understand the role of MSC in this context. We analyzed 24 patients (pts) receiving MSC for GvHD in our Unit between 2009 and 2011. We recorded viral reactivations as measured in whole blood with polymerase chain reaction for 100 days following MSC administration. In patients with a documented viral reactivation in the first 3 days following MSCs infusion the frequency of virus-specific IFNgamma-producing cells was determined through enzyme-linked immunospot assay. In our cohort of patients viral reactivation after MSC infusion occurred in 45% of the cases, which did not significantly differ from the incidence in a historical cohort of patients affected by steroid resistant GvHD and treated with conventional immunosuppression. No patient presented severe form of infection. Two cases could be checked for immunological response to viral stimulus and demonstrated virus specific T-cytotoxic lymphocyte activity. In our experience MSC infusion did not prove to trigger more frequent or severer viral reactivations in the post transplantation setting.

## 1. Introduction

Mesenchymal stromal cells (MSCs) are multipotent cells endowed with several immunomodulatory properties. Firstly isolated from human bone marrow and characterized by their ability to self-renew and differentiate into mesodermic tissues, in the last decade their immunological potential has been widely exploited in the attempt to treat inflammatory, autoimmune, and alloimmune diseases [1]. Many groups have focused their attention on the use of MSC to manage graft versus host disease (GvHD) in stem-cell-transplanted patients after the first report of clinical success was documented by Le Blanc et al. in 2004 [2].

Recent studies *in vitro* and in mouse models [3–6] have demonstrated that MSCs exert a pleiotropic immune suppressive action on ongoing immune alloreaction. Previous considerations by several groups have established that MSCs inhibit T-cell proliferation in response to alloantigens and nonspecific mitogens. This process is thought to be mediated both by the secretion of soluble factors, such as indoleamine 2,3 dioxygenase, HLA-G, prostaglandin E2, and nitric oxide and by cell-to-cell contact. MSCs are able to inhibit T- and B-cell proliferation and to impair NK and dendritic cell activity [7]. Interestingly, *in vitro* studies demonstrated that MSCs strongly suppress alloantigen induced T-cell responses without interfering with the antiviral T-cell activity [8, 9]. Moreover, it has been demonstrated that the ability of MSC to inhibit T-cell alloresponse is independent from the major histocompatibility complex [10].

As viral complications in immunocompromised hosts affected by resistant GvHD still represent a major clinical concern [11], we tried to understand if the immunosuppressive activity exerted by MSC upon *in vivo* infusion could have some impact on the risk for viral reactivations and on the correct mounting of antiviral immune responses.

The present report analyzes the risk of viral infection for cytomegalovirus (CMV), Epstein Barr virus (EBV), and adenovirus (ADV) in a cohort of patients infused with bone-marrow-derived third-party MSC, expanded with platelet lysate (PL) under Good Manufacturing Practices (GMP) conditions, as previously described in details by our group [12].

## 2. Patients and Methods

All patients received MSC for steroid resistant GvHD at two partner institutions (Ospedale San Gerardo, Monza and Ospedali Riuniti, Bergamo) from July 2009 to December 2011 and were monitored twice a week for CMV, EBV, or ADV reactivation, as measured by Polymerase Chain Reaction (PCR) assay in whole blood. Viral reactivation was defined as evidence of viral load ≥1000 copies/mL in peripheral blood. Only reactivations occurring for the first time after MSC infusion were documented and patients already receiving antiviral treatment at the time of MSC infusion were excluded from the analysis. For the purpose of the present paper, viral detections occurring between day 0 and day +100 after MSC infusion were recorded. According to local policies patients received or did not therapy with Ganciclovir or Foscavir, antiCD20moAb, or Cidofovir, respectively for CMV, EBV, and ADV.

Any patient was analyzed in search for symptoms of viral overt disease if presenting with fever or organ involvement associated with viral reactivation.

In order to allow a retrospective comparison, all patients receiving allogeneic stem cell transplantation between January 2007 and December 2008 were analyzed, and those who had developed viral reactivations after steroid resistant GvHD, but had not received MSC, were considered as control group.

Type of transplantation, T-cell depletion, conditioning regimen, and number of immunosuppressive lines administered at the time of viral reactivation were recorded, as well as GvHD grading.

In 2 patients reactivating CMV, the frequency of virus-specific cells, secreting IFN-*γ* in response to a cocktail of CMV-specific peptides [13] was measured by ELISPOT assay (EBioscience, San Diego CA, USA), before and at different time points after MSC infusion.

MSCs were obtained from third-party donors after expansion with PL, as elsewhere described [12]. Patients received MSC after giving informed consent and being registered in a phase I, bicentric, prospective trial, which had been approved by local and national authorities. Each MSC infusion aimed at delivering a median dose of 1 × 10^6^/kg body weight of the recipient, and a minimum of 2 infusions were given to each patient.

## 3. Results

### 3.1. Clinical Monitoring

From July 2009 till December 2011, 24 patients received MSCs on top of the ongoing immunosuppressive therapy to treat steroid resistant GvHD. Of these, 11 (45%) developed a viral reactivation for the first time after MSC infusion. Other 2 (8.3%) patients had developed viral reactivation before MSC infusion and were not analyzed in detail for the purpose of the present study.

All analyzed patients received an allogeneic transplantation for malignant (10 cases) or nonmalignant disease (1 case). Patient number 4 and 14 received a graft from a mismatched donor, whereas all other patients received the graft from either matched related [2] or unrelated [7] donors. Among our cohort of patients 9 received a fully myeloablative conditioning, in 4 cases TBI based and 2 got a reduced intensity conditioning according to local policies. No one of the analyzed patients received a T-cell depleted graft, but 9 patients received *in vivo* T-cell depletion as part of GvHD prophylactic regimen (Table 1). All the described patients had suffered acute GvHD grade II to IV involving 1 (6 cases) or more organs (5 cases). All patients were receiving immunosuppressive treatment at the time of viral reactivation. Seven out of 11 patients developed a reactivation from a single viral agent (Table 2).

CMV was the most common pathogen occurring in 4 seropositive patients who received seronegative graft, as well as in 3 patients who showed a more favorable serology pattern (donor and recipient CMV IgG positive). All patients developing CMV infection were treated with Ganciclovir or Foscavir according to local guidelines. Two patients (UPN #14 and 16) received a combined Ganciclovir and Foscavir treatment because of a CMV-related colitis, documented through gut biopsies. Both patients had developed gut GvHD. Another patient (UPN #10) switched to Foscavir treatment to avoid bone marrow toxicity after prolonged Ganciclovir exposure.

EBV positivity was detected in 6 patients. Four of them were treated with anti CD20 monoclonal antibodies, but none of the considered patients developed signs or symptoms of EBV-related posttransplant lymphoproliferative disorder (PTLD).

ADV reactivation occurred in 3 patients and was documented in stool as well as in blood, whilst in one case (UPN #16) gut biopsy also revealed ADV positivity. GvHD had involved gut in all three patients. All of them successfully received treatment with Cidofovir.

CMV was the most rapid raising infection in our cohort with a median time of appearance after the first MSC infusion of 2 days (range 1 to 31 days) followed by ADV (median 7 days, range 3 to 40 days) and by EBV (median 21 days, range 1 to 100). Overall, the viral reactivations occurred at a median time of 17 days after the beginning of steroid therapy in these patients.

All the described patients received multiple MSC infusions (range 2 to 8), and viral reactivation was detected in 3 cases after the first infusion and in 8 cases after further infusions. None of the patients in our cohort died for viral-related causes. Overall survival in this cohort of patients was 45,5% with a median followup of 20 months after MSC infusion (range 6 to 31 months). Concerning MSC and GvHD treatment, 2 patients of this cohort presented a complete response to treatment, 4 a partial response, 3 did not respond to MSC treatment, and in 2 cases response to MSC was not evaluable because of death before day + 28 after the last MSC infusion, which represented the evaluation time point according to the present protocol.

### 3.2. Immunological Monitoring

**Figure 1:**
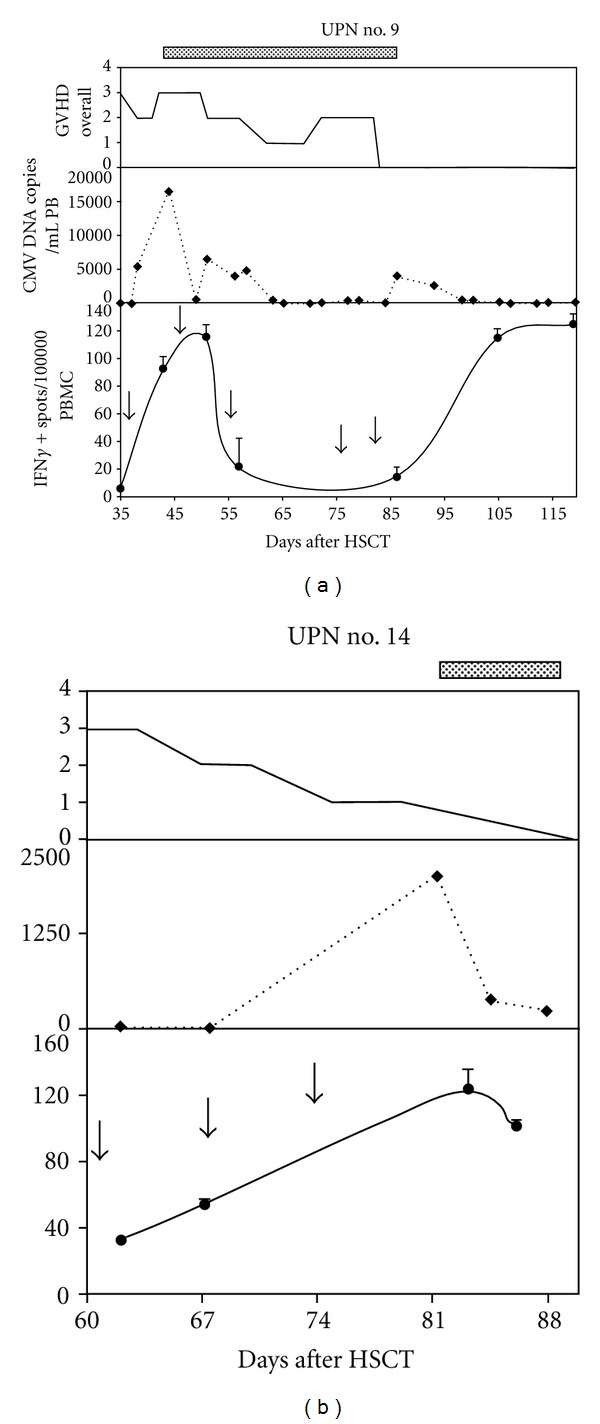
Frequency of CMV-specific T cells, secreting interferon gamma (IFN-*γ*) in response to a cocktail of CMV-specific peptides, as measured by ELISPOT assay in two patients reactivating CMV after having received mesenchymal stem cells (MSCs) for the treatment of steroid refractory graft versus host disease. Dotted lines on top of the panels indicate Ganciclovir administration. In the upper panel overall GvHD grading according to NIH criteria is shown. In the central panel Cytomegalovirus (CMV) reactivation trend as measured in copies/milliliter of peripheral blood (PB) is shown. In the lower panel the number of CMV-specific IFN-*γ* producing cells in relation to MSC infusions as measured in IFN-*γ* spots/100.000 peripheral blood-mononucleated cells (PBMC) is shown. Black arrows indicate MSC infusions.

Trying to understand if, upon *in vivo* infusion, MSC could influence virus-specific T-cell-mediated immune responses, we evaluated by ELISPOT assays the frequency of virus-specific T cells circulating in the peripheral blood (PB) of 2 patients experiencing CMV reactivation soon after MSC infusion (UPN #9 and 14). Both patients showed an increase of CMV-specific IFN-*γ* producing cells in the PB along with CMV reactivation, despite the concomitant infusion of multiple MSC doses (Figure 1). In particular, in UPN #9 CMV-specific cells increased 18 times at day 44 after HSCT, upon CMV reactivation (16700 CMV DNA copies/mL of PB), compared to day 35 (preinfection levels). Virus-specific cells then started to decrease along with the resolution of CMV infection and raised again upon a second episode of CMV reactivation at day 86. Similarly, in UPN #14, CMV-specific T cells increased up to 4 times along with CMV reactivation at day 80 after HSCT.

### 3.3. Comparison with Historical Cohort of Patients

Between January 2007 and December 2008, 52 patients received allogeneic stem cell transplantation at San Gerardo Hospital (Monza). 14 of them received stem cell from an HLA identical related donor, 4 from a related mismatched donor, and the remaining patients from an HLA-matched-unrelated donor. Six patients received stem cells from cord blood, 42 from bone marrow, and 4 from peripheral stem cells. Only one graft was T-cell depleted. GvHD prophylaxis included ATG in 35 cases. Among this group of patients 20 developed acute-or chronic-steroid-resistant GvHD of grade II or severer. All of them received further lines treatment after steroid according to GvHD and patient characteristics (2 to 6 lines of treatment). 10 of those patients (50%) presented with viral reactivation after GvHD onset. 5 of them presented with a combined EBV and CMV reactivation, one patient presented with isolated EBV, and 4 with isolated CMV reactivation. None of these patients exhibited a PTLD nor CMV disease. Median time from start of GvHD treatment to viral reactivation was 13 days (range 2 to 39). Eight of these 10 patients are alive with a median followup of 48 months from HSCT (range 34 to 62 months), none of those patients died from viral-related causes.

## 4. Discussion

MSC proved to be a useful tool in managing GvHD resistant to conventional treatment. According to different published observations, the response rate varies from 52 to 70% and differs between adults and children [14, 15].

In spite of the clinical evidence of efficacy, it is still unrevealed how these cells are able to tune the alloreactivity in the recipient. We recently demonstrated that MSCs, upon infusion, are able to convert an inflammatory environment to a more physiological one, both at the cellular level, promoting the increase of T-reg cells circulating in the peripheral blood and at the molecular level, diminishing the concentration of inflammatory cytokines [16]. However, concerns are recently arising about the possibility that MSCs could, through their immunosuppressive action, impair anti-viral T-cell responses [17].

The question whether these cells could also influence other immunological activities of the effector T cells, namely antiviral and antibacterial capacity, was firstly addressed by Karlsson et al. [9]. In a very detailed series of assessments, they documented in 2 different patients, that effector function of virus-specific T cells could be retained after MSC infusions. No other clinical detailed data are available, to our knowledge, to support this initial observation. Meisel et al. [18] reported *in vitro* antimicrobial activity of MSC, without further assessment on antiviral properties. The only phase III randomized study, which was so far conducted on MSC administration, also reported a general note on unaltered infection rate between the experimental and the control group of the cohort [19].

A recent report [17] by Ringdén et al. describes a cohort of 31 patients treated with MSC infusion for aGVHD or hemorrhagic cystitis describing a high incidence of CMV reactivations and mild disease (31% of CMV-related disease). This high frequency was expected in patients with steroid-refractory GVHD, which represents a high-risk group for developing viral diseases. Accordingly, they reported that CMV disease occurred in a similar number of patients in the same cohort before MSC infusion.

In our described population the overall viral reactivation rate after MSC infusion was 45%, with only 8.3% of CMV-related disease (i.e., colitis). In our cohort, as well as in the one reported by Ringdén, patients were receiving already at least one line of immunosuppression at the time of MSC administration. For this reason it is impossible to restrict the significance of our data to MSC administration. It is anyhow relevant to underline that our viral infectious rate was similar to what reported by other groups in patients receiving experimental treatments for resistant GvHD other than MSC [20], and that all patients in our cohort were able to overcome the viral reactivation with conventional treatment, without reporting any viral-infection-related death. Moreover, a retrospective comparison with a group of patients who developed viral reactivation after steroid refractory GvHD and did not receive MSC treatment also revealed similar percentage of viral reactivations (50% versus 45% in the MSC treated group) and similar latency between the start of the steroid therapy and the viral reactivation.

To support our clinical data, we demonstrated, through immunological monitoring, how patients exposed to CMV reactivation after MSC infusions were able to mount a physiological antiviral immune response. It is important to note that both analyzed patients were at the same time responding to MSC infusions, as demonstrated by the progressive attenuation of the GVHD overall clinical score. In particular, the observation that every episode of viral reactivation is accompanied by an increase of CMV-specific T cells and that this increase, along with the adopted antiviral drug therapy, results in the clearance of the infection, demonstrates that MSCs do not interfere with the antiviral response. These results, in line with data already obtained by other authors, support the concept that MSCs are able, upon *in vivo* infusion, to suppress GVHD promoting alloantigen induced T-cell responses sparing somehow virus-specific immune responses [9]. This data holds true in our patients even after repeated expositions to MSC, thus allowing a safe repeated use of these cells.

The present paper confirms safety data on the possible use of MSC in transplanted patients. Our experience suggests that no augmented risk of viral reactivation or disease is present for MSC-infused patients, and that those who develop viral reactivation do not present more severe course of disease since the capacity to expand virus-specific T cells is not impaired.

## Figures and Tables

**Table 1:** Patients characteristics.

UPN	Diagnosis	Conditioning regimen	GvHD prophylaxis	GvHD	IS treatment at viral reactivation
1	ALL	TBI + VP16	CSA + MTX + ATG	Acute skin grade II	CSA
4	ALL	TBI + VP16	CSA + MTX + ATG	Acute skin + liver grade III	mPDN, MMF
7	MDS	BU + CY + MEL	CSA + MTX + ATG	Acute gut grade III	mPDN, MMF
9	MNGIE	BU + FLU	CSA + MTX + ATG	Acute gut grade III	mPDN
10	ALL	TBI + VP16	CSA + MTX + ATG	Acute skin grade II	mPDN, CSA
11	AML	BU + CY + MEL	CSA + MTX + ATG	Acute skin + gut + liver grade IV	mPDN, MMF, ETANERCEPT
13	ALL	TREO + FLU	CSA + MTX + ATG	Acute skin grade II	CSA
14	ALL	TT + BU + FLU	CSA + MMF + EDX	Acute gut + liver grade III	mPDN, CSA
15	AML	TREO + FLU	CSA + MTX	Acute Skin + gut + liver grade III	mPDN, CSA
16	ALL	TBI + CY	CSA + mPDN + ATG	Acute gut + liver grade IV	CSA, mPDN, ECP, PENTOSTATIN IMATINIB, CAMPATH
17	AML	BU + CY	CSA + mPDN	Acute gut grade IV	mPDN, CSA

IS: immunosuppression, ALL: acute lymphoblastic leukemia, AML: acute myeloid leukemia, MNGIE: mitocondrial neurogastrointestinal encephalomyopathy, TBI: total body irradiation, BU: busulfan, CY: cyclophosphamyde, MEL: melphalan, TREO: treosulfan, FLU: fludarabine, TT: Thiotepa, CSA: cyclosporine A, MTX: methotrexate, ATG: antithymocyte globulin, mPDN: methylprednisolon, MMF: mofetilmycophenolate, ECP: extracorporeal photopheresis. GvHD is graded according to NIH criteria.

**Table 2:** Viral reactivations details.

UPN	Virus	Sample	Days from MSC infusion	Treatment	Outcome
1	EBV	Blood	100	Rituximab	Alive, no GvHD
4	CMV EBV	Blood Blood	13 40	Ganciclovir Rituximab	Alive, no GvHD
7	ADV	Blood + stool	7	Cidofovir	Alive, no GvHD
9	CMV	Blood	1	Ganciclovir	Died on day +456 from SCT from sepsis
10	CMV	Blood	2	Ganciclovir then Foscavir	Alive, no GvHD
11	ADV	Blood + stool	3	Cidofovir	Died on day +90 from SCT from GvHD
13	EBV	Blood	44	Rituximab	Alive, no GvHD
14	EBV CMV	Blood Blood + gut biopsy	1 7	Rituximab Ganciclovir+ Foscavir	Died on day +129 from SCT from sepsis
15	CMV EBV	Blood Blood	1 1	Ganciclovir None	Died on day +168 from SCT from septic shock
16	EBV CMV ADV	Blood Blood + gut biopsy Blood + gut biopsy	1 1 40	None Ganciclovir + Foscavir Cidofovir	Died on day +225 from SCT from GvHD
17	CMV	Blood	31	Ganciclovir	Died on day +136 from SCT from disease recurrence

EBV: Epstein Barr Virus, CMV: Cytomegalovirus, ADV: Adenovirus, GvHD: Graft versus Host Disease, and SCT: stem cellstransplantation.
